# Isolation, Identification, and Drug Susceptibility Testing of the Pathogen Causing Perforation Disease in Giant Spiny Frog Tadpoles (*Quasipaa spinosa*)

**DOI:** 10.3390/microorganisms14051016

**Published:** 2026-04-30

**Authors:** Qinglian Wu, Xiandong Xu, Jianqin Li, Han Qiu, Huanhuan Huo, Mo Peng, Chungen Wen, Gang Yang

**Affiliations:** 1 Key Laboratory for Aquatic Germplasm Innovation and Utilization of Jiangxi Province, School of Life Science, Nanchang University, Nanchang 330031, China; 2 School of Animal Science and Technology, Jiangxi Agricultural University, Nanchang 330045, China

**Keywords:** giant spiny frog, tadpole, perforation disease, *Pseudomonas* sp., drug susceptibility testing

## Abstract

A pathogenic bacterium strain, LBK2, was isolated from giant spiny frog (*Quasipaa spinosa*) tadpoles infected with perforation disease in this study. Pathogenic strain LBK2, a Gram-negative bacterium with a certain degree of infectivity, was demonstrated to cause anorexia, lethargy, epidermal necrosis, and abdominal perforation in tadpoles in artificial infection experiments. The identification results of 16S rDNA gene sequencing showed that pathogenic strain LBK2 was identified as *Pseudomonas* sp. Virulence gene identification displayed that strain LBK2 carried three virulence genes: *aer*, *epr*, and *fla*. Finally, the antibiotic susceptibility testing of 11 antibiotics suggested that strain LBK2 was highly sensitive to nine antibiotics, including chloramphenicol, enrofloxacin, and rifampicin, but was resistant to erythromycin, sulfamethoxazole/trimethoprim, and low-concentration trichloroisocyanuric acid. This study determined the pathogenicity of *Pseudomonas* sp. to giant spiny frog tadpoles based on histopathological analysis and virulence factor carriage, and the drug susceptibility testing further provided a scientific basis for the selection of drugs in the prevention and treatment of abdominal perforation disease in giant spiny frog tadpoles.

## 1. Introduction

The giant spiny frog, scientifically known as *Quasipaa spinosa*, is also commonly referred to as the stone frog in China. It belongs to the Amphibia class, the Anura order, and the Ranidae family. This species is primarily distributed in the hilly and mountainous regions of southern China, encompassing provinces such as Jiangxi, Fujian, Zhejiang, Hunan, Anhui, Guangxi, and others [1,2]. The giant spiny frog boasts a large and robust body, resembling the black-spotted frog or tiger-striped frog, but with varying body colors. For instance, some individuals exhibit a black dorsal surface adorned with a golden midline. It is straightforward to distinguish the males and females of the giant spiny frog. The male individuals possess sturdy forelimbs with narrow, elongated warts interspersed with small, round warts and, upon sexual maturity, develop black, spine-like projections across their chests. In contrast, the female individuals have less developed forelimbs, lack the narrow, elongated warts and spine-like projections on their chests, and possess a smooth, white abdomen [3,4]. In recent years, the giant spiny frog has emerged as an important species for aquaculture in China. Taking Jiangxi Province as an example, the giant spiny frog aquaculture industry has flourished due to its unique geographical and ecological advantages. In 2022, the total aquaculture area for giant spiny frogs in Jiangxi was 72.13 hectares, and its production output reached 322.4 tons, generating an economic value exceeding 1.52 million dollars [5]. During the breeding process of giant spiny frogs, the tadpole cultivation represents a crucial stage and restricts the rapid development of this industry, as the survival rate of tadpoles is low. Ensuring good water quality and maintaining appropriate water temperatures are fundamental to the healthy growth of tadpoles. Additionally, proper stocking densities and rational feeding practices significantly impact tadpole development [6]. However, the continuous expansion of the breeding scale of the giant spiny frog and the blind pursuit of increased production have led to a growing demand for seedlings, and thus the density of tadpole stocking has been constantly rising, resulting in an increasing frequency of various diseases year by year [7]. A large number of previous studies have confirmed that disease prevention and control of frog tadpoles (especially *Quasipaa spinosa* tadpoles) constitute a core challenge in the breeding process. Common pathogenic pathogens are mainly categorized into two groups: parasites and bacteria, with distinct symptoms induced by different pathogens. In terms of parasitic pathogens, in addition to *Hexamita* spp. [8] and *Trichodina* [9], other prevalent parasitic pathogens in tadpole culture include *Ichthyophthirius*, *Chilodonella*, and *Epistylis*. Among these, *Trichodina* commonly parasitizes the gills and body surface of tadpoles, damaging gill filament structures and impairing respiratory function; severe infestations can lead to tadpole death by suffocation. *Hexamita*, by contrast, mainly colonizes the intestinal tract, triggering enteritis, diarrhea, emaciation, and reduced immunity in tadpoles [10]. Regarding bacterial pathogens, pathogenic isolates frequently recovered from diseased *Quasipaa spinosa* tadpoles include not only *Aeromonas hydrophila* [11], Klebsiella pneumoniae [12], *Bacillus cereus* [13], and *Citrobacter braakii* [14], but also *Escherichia coli*, *Aeromonas sobria*, and *Pseudomonas fluorescens*. As a typical opportunistic pathogen, *Aeromonas hydrophila* proliferates rapidly under deteriorated rearing conditions or when tadpole immunity declines, causing acute diseases such as hemorrhagic septicemia and gill rot with extremely high mortality rates [15,16]. Furthermore, saprolegniasis caused by fungi of the genus *Saprolegnia* is a common disease during the overwintering period of *Quasipaa spinosa* tadpoles. It frequently breaks out under low-temperature and high-humidity conditions, resulting in white cotton-like hyphae on the tadpole body surface, followed by skin ulceration, organ failure, and eventual mortality [17].

The pathogenicity of a pathogen is determined by multiple factors, including the biological state of the host, the environment, and the virulence of the pathogen. The virulence of a pathogen often results from the synergistic action of multiple virulence genes [18]. For instance, Wang Wen-Hui et al. detected four virulence genes, namely exoT, exoY, exoS and exoU, in Pseudomonas aeruginosa, which are the key determinants of its virulence [19,20]. Meanwhile, existing research has shown that *Pseudomonas fluorescens* contains multiple virulence factors, which can act individually or in combination, resulting in a complex pathogenic mechanism. Generally, it is believed that the virulence of *Pseudomonas fluorescens* is closely related to the types and quantities of virulence genes it carries. *Pseudomonas fluorescens* carries six virulence genes, including fur, hly, hemO, fha, pspB, and tfeR, indicating that it has hemolytic activity and protease activity [21]. The fla gene, among others, enables the pathogen to move, infect, and adhere within the host [22]. The aer gene encodes siderophores that compete with the host for iron ions, affecting the host’s nutrient metabolism [23]. The epr gene encodes the production of exotoxins, which are secreted by bacteria during growth and reproduction, causing harm to organisms [24].

Perforating disease was commonly reported in *Pelodiscus sinensis* [25] and *Cyprinus carpio* [26], and it has also been observed on the tadpoles of the giant spiny frog. This disease is highly contagious in *Pelodiscus sinensis*, with a wide epidemic range, long duration, and high incidence rate, and can lead to mass mortality, causing a significant economic loss to the turtle aquaculture industry [27,28]. Similarly, this disease is highly contagious and can cause anorexia, drowsiness, skin rot, and abdominal perforation in tadpoles. The phenomenon of tadpole mortality due to perforating disease is not uncommon in practical production, and the insufficient supply of seedlings will restrict the rapid development of the giant spiny frog industry. Evidence from the studies in *P. sinensis* and *C. carpio* suggested that the perforating disease was caused by the infection of pathogenic bacteria, including *Aeromonas hydrophila* [25,26] and *Sphingomonas paucimobilis* [29]. Notably, species of the genus Pseudomonas have also been frequently isolated from aquatic animals with ulcerative and perforative symptoms and are regarded as important opportunistic or primary pathogens in fish, amphibians, and reptiles [30,31,32]. The literature and clinical studies have shown that A. hydrophila is highly sensitive to amikacin, tobramycin, norfloxacin, and gentamicin, while *S. paucimobilis* is sensitive to sulbactam sodium, piperacillin, tazobactam, ceftazidime, ceftriaxone sodium, cefepime, imipenem, meropenem, tobramycin, levofloxacin, and trimethoprim [27,28,29,30]. Although various bacterial diseases have been documented in amphibians, the etiology of perforation disease in giant spiny frog (*Quasipaa spinosa*) tadpoles—a condition responsible for substantial economic losses—remains poorly understood. To date, the causative pathogen, its virulence mechanisms, and evidence-based control strategies have not been established, resulting in empirical and often ineffective antibiotic use in aquaculture practice.

The scientific significance of this study is threefold. First, to the best of our knowledge, this is the first study to systematically identify and validate *Pseudomonas* sp. LBK2 as the primary etiological agent of perforation disease in *Q. spinosa* tadpoles, fulfilling Koch’s postulates. Second, we characterized the virulence gene profile of the pathogen, providing molecular insights into the pathogenesis of abdominal perforation and other clinical manifestations. Third, the antibiotic susceptibility profile determined in this study offers a scientific basis for the rational selection of therapeutic agents, thereby contributing to the sustainable development of the giant spiny frog aquaculture industry.

## 2. Materials and Methods

### 2.1. Clinical Symptoms

Clinical diagnosis was performed based on typical macroscopic lesions such as epidermal necrosis and abdominal perforation, combined with farm epidemiological data. Diseased lesion tissues were collected aseptically for subsequent bacterial isolation and identification.

### 2.2. Isolation and Purification of Pathogenic Bacteria

In November 2023, a disease outbreak occurred among tadpoles at a giant spiny frog farm in Fuzhou City, Jiangxi Province, China. The affected tadpoles exhibited similar symptoms, including lethargy, anorexia, skin surface decay, and abdominal ulceration with perforation. They exhibited symptoms of sluggishness, poor appetite, skin surface decay, and abdominal ulceration with perforation. The water temperature for the breeding environment of the giant spiny frog tadpoles was maintained at 24 °C. Tissue sections were prepared from the affected areas of the tadpoles and observed under an optical microscope, revealing no signs of parasitic infection. Under sterile conditions, the skin surface of the diseased tadpoles was gently wiped with 75% alcohol, and the affected tissue was excised and placed in a centrifuge tube containing tissue preservation solution (glycerol/saline = 1:1). Three to four sterile grinding beads were added, and the tissue was homogenized at room temperature with a programmed setting of 70 Hz for 45 s, followed by a 15 s interval, repeated ten times. After homogenization, the homogenate was diluted 10-fold and 100-fold, respectively, and spread onto pre-prepared Luria–Bertani (LB), Nutrient Broth (NB) and Brain Heart Infusion (BHI) media. Six Petri dishes (three concentration gradients, one set of parallel experiments) were used for each medium type. The plates were incubated for 24 h at 24 °C. Based on morphological differences such as size, shape, edge, and gloss, different single colonies were picked from the media and purified using the streak plate method on fresh media. This process was repeated for three consecutive purifications to obtain pure bacterial strains. The obtained colonies were inoculated into liquid media for amplification, and the expanded bacterial suspension was mixed with an equal volume of 50% glycerol and stored at −20 °C. All experiments were approved by the Institutional Animal Care and Use Committee of Nanchang University (Approval number: NCULAE-20241231999).

### 2.3. Identification of Pathogenic Bacteria

#### 2.3.1. Gram Staining

The pathogenic bacteria were subjected to Gram staining for identification, following the methodology described in reference [16]. A drop of ddH_2_O was placed in the center of the slide, and a single colony from the isolated and purified bacteria was picked and smeared. The smear was first stained with crystal violet as the primary stain, followed by iodine for mordanting. It was then decolorized with 95% ethanol and counterstained with safranin. After drying, the slide was observed and photographed under an optical microscope.

#### 2.3.2. 16S rDNA Identification

A total of 50 μL of bacterial suspension was mixed with 100 μL of ddH_2_O in a 2 mL centrifuge tube and vortexed thoroughly. The mixture was heated in a water bath at 100 °C for 10 min, cooled, and then centrifuged at 12,000 rpm for 10 min. The supernatant, including bacterial DNA, was collected and stored at −20 °C for future PCR use. PCR amplification was performed using the abovementioned supernatant as a template. The universal primers for bacterial 16S rDNA were synthesized by Youkang Biotechnology Co., Ltd. (Hangzhou, China). The primer sequences are listed in Table 1. The PCR products were sent to Beijing TsingKe Biotechnology Co., Ltd. (Beijing, China) for sequencing. The sequencing results were compared with the database at the National Center for Biotechnology Information (NCBI), and a phylogenetic tree was constructed using Mega software version 12.0. The species of the pathogenic bacteria were determined through 1000 bootstrap replications to assess the confidence level.

PCR Reaction Conditions: A 50 μL reaction system was used, with each tube containing 25 μL of Taq DNA Polymerases, 15 μL of ultrapure water, 5 μL of template DNA, and 2.5 μL of each upstream and downstream primer. The PCR amplification protocol was as follows: initial denaturation at 95 °C for 5 min, followed by 30 cycles of denaturation at 95 °C for 30 s, annealing at 57 °C for 45 s, and extension at 72 °C for 90 s. A final extension step was performed at 72 °C for 10 min. The PCR products were analyzed by 1% agarose gel electrophoresis.

### 2.4. Virulence Gene Detection

Thirteen common bacterial virulence genes, including ompAII, hly, Aer, lip, lafA, ahp, epr, Alt, Ast, gcaT, ompAI, fla, and Act, were selected for detection [17,18]. The primer sequences are listed in Table 1. PCR amplification was performed using the DNA of the LBK2 strain as template, and the PCR products were sent to Beijing TsingKe Biotechnology Co., Ltd. (Beijing, China) for sequencing.

PCR Reaction Conditions: A 50 μL reaction system was used, with each tube containing 25 μL of Taq DNA polymerase, 15 μL of ddH_2_O, 5 μL of template DNA, and 2.5 μL of each upstream and downstream primer. The PCR amplification protocol was as follows: initial denaturation at 95 °C for 2 min, followed by 35 cycles of denaturation at 95 °C for 30 s, annealing at 56 °C for 45 s, and extension at 72 °C for 60 s. A final extension step was performed at 72 °C for 10 min. The PCR products were analyzed by 1% agarose gel electrophoresis.

### 2.5. Antibiotic Susceptibility Test

Different amounts of trichloroisocyanuric acid (0.1 g, 0.2 g, 0.3 g, 0.4 g) were dissolved in 50 mL of ddH_2_O by water bath heating. Circular filter papers with a diameter of 6 mm were immersed in the solutions to absorb the drug and then dried in a desiccator for 24 h before use. Antibiotic susceptibility disks were thawed at room temperature before use, having been stored at −20 °C.

The bacterial suspension was diluted to 1 × 10^6^ CFU/mL, and 100 μL of the suspension was evenly spread onto MH medium. Sterile tweezers were used to apply the antibiotic disks onto the surface of the medium. The prepared medium was then inverted and incubated at 37 °C for 24 h. The diameter of the inhibition zone was measured using a vernier caliper (in triplicate). Finally, the sensitivity of the pathogen to 11 antibiotics and trichloroisocyanuric acid was determined based on the standard for antimicrobial susceptibility testing [31].

### 2.6. Artificial Infection Tests and LD_50_ Determination

The LBK2 strain was subjected to artificial re-infection experiments to determine its pathogenicity. The artificial infection test was designed with two experimental groups (feeding group and immersion group) and two control groups. For the feeding group, healthy giant spiny frog tadpoles were fed with bacterial concentrations of 1 × 10^4^, 1 × 10^5^, 1 × 10^6^, 1 × 10^7^, and 1 × 10^8^ CFU/mL of the LBK2 strain, and their control group was immersed in an equal volume of sterile saline solution. Healthy giant spiny frog tadpoles were selected and temporarily reared at a water temperature of 23 °C for 7 days. They were then grouped and cultured in 80 cm × 50 cm × 20 cm glass aquariums with normal feeding and a continuous drip system (using well water that had been left to stand for 24 h, with a water volume of 5 L and a temperature maintained at 23 °C). Each group contained 10 tadpoles, divided into the feeding and immersion groups. The feeding group was fed daily with feed mixed evenly with 200 μL of LBK2 bacterial suspension, while the immersion group had 400 μL of LBK2 bacterial suspension added to their water. The health status of the tadpoles was recorded daily for two weeks.

### 2.7. Statistical Methods and Construction of 16S rDNA Sequence Phylogenetic Tree

#### 2.7.1. Statistical Method for LD_50_ Calculation

The median lethal dose (LD_50_) was calculated using SPSS 26.0 software (IBM Corp., Armonk, NY, USA) with Karber’s method as the statistical model. Prior to statistical analysis, the experimental data were subjected to a normality test and homogeneity of variance test to ensure the validity of the analysis. LD_50_ values and their corresponding 95% confidence intervals were computed using Karber’s formula: LD_50_ = lg^−1^[Xm − i(ΣP − 0.5)], where Xm represents the logarithm of the highest dose, i is the logarithmic difference between adjacent dose groups, and P denotes the mortality rate of each group.

#### 2.7.2. Construction of 16S rDNA Phylogenetic Tree

For the phylogenetic analysis of 16S rDNA sequences, the obtained 16S rDNA sequences of the isolated strains were first subjected to BLAST analysis (2.17.0) in the NCBI GenBank database, and homologous sequences with a similarity of ≥97% were retrieved using the BLAST tool. Multiple sequence alignment was performed with ClustalX 2.1 software under default parameters. The phylogenetic tree was constructed using MEGA 11.0 software, adopting the Neighbor-Joining (NJ) algorithm as the tree-building method and the Kimura 2-parameter model for evolutionary distance calculation. To verify the reliability of the phylogenetic tree, 1000 bootstrap replicates were conducted, and the bootstrap values were labeled at the branch nodes.

## 3. Results

### 3.1. Symptoms of Diseased Tadpoles

**Figure 1:**
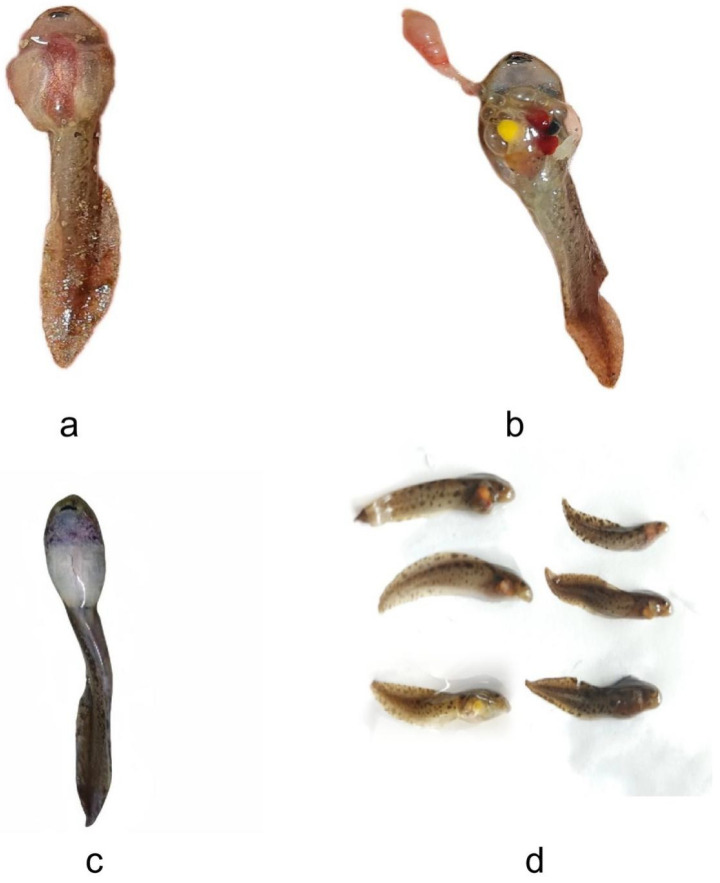
Schematic diagram of diseased tadpoles and healthy tadpoles. Note: (**a**,**b**) The abdomen of the diseased tadpole shows visible red swelling erosion; (**c**) the skin of the diseased tadpole is damaged, the internal tissue is exposed, and the abdominal tissue is prominent with yellow plaques; (**d**) the healthy tadpole.

The abdomens of diseased tadpoles exhibited visible redness, swelling, and erosion (Figure 1b), with damaged skin, exposed internal tissues, protruding abdominal tissues, and yellow patches (Figure 1a,c). Behaviorally, they displayed sluggish movement and loss of appetite. In contrast, healthy tadpoles had smooth skin surfaces, no abdominal redness or swelling (Figure 1d), swam rapidly, and showed a strong attraction to fed bait.

### 3.2. Pathogen Identification

#### 3.2.1. Morphological Observation of Pathogens and Gram Staining

Pathogenic bacteria were isolated and purified from the lesion sites of diseased tadpoles. A total of 9 different species of bacteria were successfully isolated and initially identified in this study, including *Pseudomonas* spp., *Elizabethkingia* spp., *Bacillus cereus*, etc. Dominant strains were selected by observing colony morphology, and physiological and biochemical identification as well as 16S rDNA sequencing were sequentially performed on the candidate strains to clarify the taxonomic status of each strain. All isolated strains were expanded in culture and subjected to regression infection experiments, and the pathogenic strain causing perforation disease in *Quasipaa spinosa* tadpoles was finally identified. This strain, designated LBK2, formed rough, convex, circular colonies on LB agar medium (Figure S1a). On NB and BHI media, the colonies exhibited similar morphology, with larger, smooth, circular colonies (Figure S1b,c). Further purification of the LBK2 strain was performed (Figure S1d).

#### 3.2.2. 16S rDNA Identification and Phylogenetic Analysis

**Figure 2:**
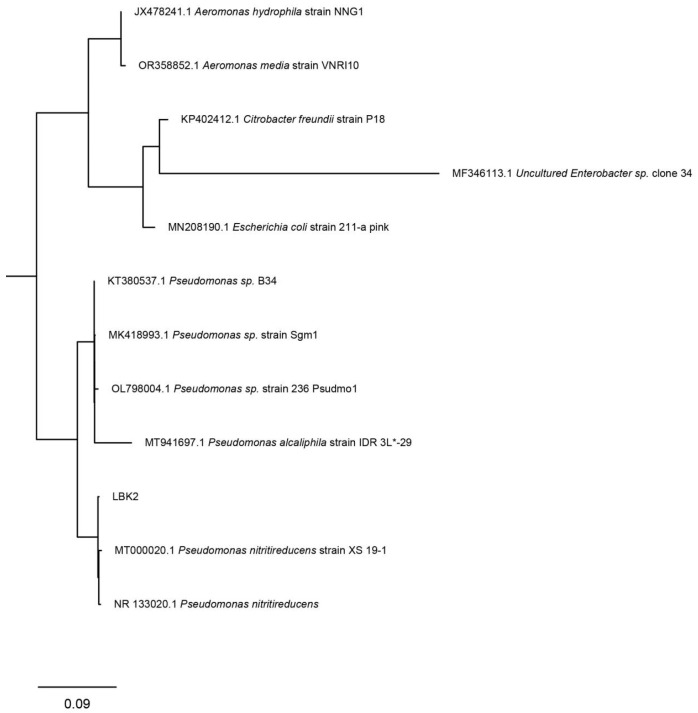
Phylogenetic tree based on strain LBK2.

The 16S rDNA gene of the LBK2 strain was amplified using universal bacterial 16S rDNA primers and detected by agarose gel electrophoresis, yielding a clear target band of approximately 1500 bp (Figure S2). BLAST analysis of the sequencing results against NCBI databases showed over 99% similarity to Pseudomonas sp. The nucleotide sequence of the 16S rDNA gene of LBK2 has been uploaded to NCBI under accession number PQ316048. Phylogenetic analysis revealed that the isolated strain clustered with *Pseudomonas* sp., indicating a close evolutionary relationship (Figure 2).

### 3.3. Virulence Gene Detection

**Figure 3:**
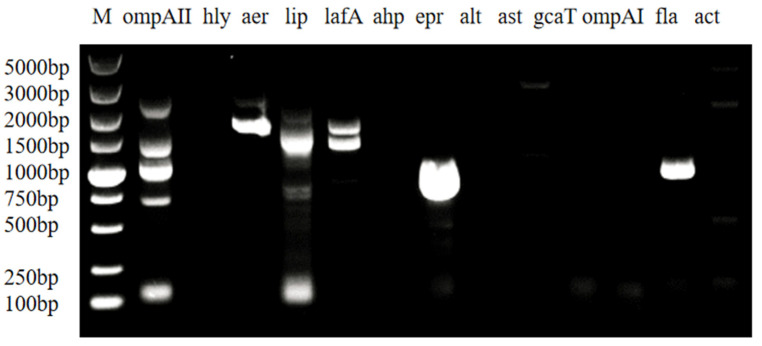
Toxicity gene detection results.

The virulence gene detection results (Figure 3) indicated that the strain carried three virulence genes: aer, epr, and fla.

### 3.4. Antibiotic Susceptibility Test

The antibiotic susceptibility test was performed on the LBK2 strain after 24 h of culture, following CLSI standards to determine its susceptibility to various antibiotics. Partial results are shown in Figure S3, and the sizes of inhibition zones are listed in Table 2. The strain was highly susceptibile to polymyxin, neomycin, florfenicol, amikacin, ampicillin, doxycycline, enrofloxacin, ceftriaxone, and rifampicin. However, it showed resistance to erythromycin, trimethoprim-sulfamethoxazole, and low concentrations of trichloroisocyanuric acid.

**Table 2:** Drug Sensitivity Test Results.

Antibiotics	Antibiotic Content	R	I	S	Measured Inhibition Zone Diameter (mm, Three Replicates)	Susceptibility
Trichloroisocyanuric Acid	0.1 g/50 mL	≤15	16~21	≥22	10.34	R
Trichloroisocyanuric Acid	0.2 g/50 mL	≤15	16~21	≥22	12.78	R
Trichloroisocyanuric Acid	0.3 g/50 mL	≤15	16~21	≥22	17.01	I
Trichloroisocyanuric Acid	0.4 g/50 mL	≤15	16~21	≥22	17.93	I
Erythromycin	15 μg/tablet	≤13	14~22	≥23	9.02	R
Compound Xinnuoming	23.75/1.25 μg/tablet	≤10	11~15	≥16	6.23	R
Polymyxin B	300 IU/tablet	≤12	13~16	≥17	13.97	I
Amikacin	30 μg/tablet	≤14	15~16	≥17	20.01	S
Neomycin	30 μg/tablet	≤12	13~14	≥15	17.54	S
Florfenicol	30 μg/tablet	≤12	13~17	≥18	22.21	S
Chloramphenicol	30 μg/tablet	≤12	13~17	≥18	21.60	S
Doxycycline	30 μg/tablet	≤14	15~18	≥19	24.03	S
Enrofloxacin	10 μg/tablet	≤18	19~21	≥22	24.46	S
Ceftriaxone	30 μg/tablet	≤13	14~20	≥21	21.34	S
Rifampicin	5 μg/tablet	≤16	17~19	≥20	25.45	S

### 3.5. Artificial Infection Tests and LD_50_ Determination

The results of the artificial infection tests showed that both feeding and immersion methods produced symptoms in tadpoles similar to those observed in sampled tadpoles infected with the strain named LBK2, including loss of appetite, abdominal perforation, bleeding, and ulceration. Compared to the feeding method, the immersion method led to earlier mortality, with deaths occurring on the first day versus the third day in the feeding group. The control groups exhibited rapid movement, good appetite, and no signs of ulceration or bleeding. Using SPSS software for statistical analysis, the LD50 values for LBK2 infection via feeding and immersion were calculated to be 6.11 × 10^6^ CFU/mL and 2.68 × 10^6^ CFU/mL respectively (Table 3 and Table 4). Furthermore, pathogenic strain LBK2 was re-isolated from the tadpoles that died during the regressive infection experiments.

**Table 3:** Pathogenicity of isolated strains on spiny breasted frog tadpoles—feeding method (number of deaths/number of experiments).

Strain Number	Control Group	1 × 10^4^	1 × 10^5^	1 × 10^6^	1 × 10^7^	1 × 10^8^	LD50 (CFU/mL)
LBK2	0/14	0/14	3/14	4/14	7/14	10/14	6.11 × 10^6^

**Table 4:** Pathogenicity of isolated strains on spiny breasted frog tadpoles—immersion method (number of deaths/number of experiments).

Strain Number	Control Group	1 × 10^4^	1 × 10^5^	1 × 10^6^	1 × 10^7^	1 × 10^8^	LD50 (CFU/mL)
LBK2	0/14	2/14	3/14	5/14	9/14	10/14	2.68 × 10^6^

## 4. Discussion

The pathogen LBK2, identified as *Pseudomonas* sp., was isolated from the tadpoles of the giant spiny frog infected with perforating disease in this study, and its infection could result in massive tadpole mortality. *Pseudomonas* sp., a Gram-negative bacterium, are strictly aerobic opportunistic pathogens, widely distributed in nature, including air, water, soil, and food [33,34,35]. This species exhibits flagellar motility, possesses a capsule, and lacks spores. They can form green or yellow-green colonies, and common species include *P. aeruginosa* [36,37], *P. putida* [38,39], and *P. fluorescens* [40]. Many *Pseudomonas* spp. are aquatic pathogens that exhibit strong infectivity towards various aquatic organisms, such as *Oreochromis mossambicus* [41], *Siniperca chuatsi* [42], *C. carpio* [43], *Andrias davidianus* [44], *Chinemys reevesii* [45], and *Larimichthys crocea* [46], posing a significant threat to the safety of the aquaculture industry. When the immune system of aquatic animals declines or their skin surface is damaged, *Pseudomonas* sp. can infect the host, leading to symptoms like anorexia, lethargy, skin ulceration, visceral enlargement with hemorrhage, and growth retardation [47]. In severe cases, it can even cause massive mortality among aquatic animals, becoming one of the serious pathogens that threaten the development of aquaculture [22]. For instance, the infection of *P. aeruginosa* led to an outbreak of mortality in *Micropterus salmoides* in a farm in Nanyang, Henan Province, China, causing severe economic losses [48]. Similarly, *P. putida* infection was reported to cause a large number of deaths in *Scophthalmus maximus* in Yantai, Shandong Province, China, concurrent with a phenomenon of ascites, hepatic congestion, and swelling of the spleen and kidneys [47].

The pathogenicity of a pathogen is determined by multiple factors, including the biological state of the host, the environment, and the virulence of the pathogen. The virulence of a pathogen often results from the synergistic action of multiple virulence genes, which can act individually or in combination, resulting in a complex pathogenic mechanism [18]. *P. fluorescens* was detected to contain multiple virulence factors, including fur, hly, hemO, fha, pspB, and tfeR [21]; however, exoT, exoY, exoS and exoU are the key determinants of virulence in *P. aeruginosa* [19,20]. In this study, *Pseudomonas* sp. LBK2 was identified to carry virulence genes such as the exotoxin protease gene (epr), flagellin structural gene (fla), and siderophore gene (aer) [49,50]. Generally, it is believed that the virulence of pathogenic bacteria is closely related to the types and quantities of virulence genes it carries. The fla gene enables the pathogen to move, infect, and adhere within the host. The aer gene encodes siderophores that compete with the host for iron ions, affecting the host’s nutrient metabolism [23]. The epr gene encodes the production of exotoxins, which are secreted by bacteria during growth and reproduction, causing harm to organisms [24]. Accordingly, these virulence genes played an important role in the infection process of giant spiny frog tadpoles by pathogen LBK2.

Research on bacterial pathogens in aquatic animals has consistently demonstrated that skin and gill tissues serve as highly efficient portals of entry for immersion-based infections. Studies on *Pseudomonas plecoglossicida* in ayu (*Plecoglossus altivelis*) using quantitative PCR revealed that the bacterium was detected in skin and gill tissues as early as 1–3 h post-immersion, with subsequent dissemination to internal organs (liver, spleen, and kidney) by 6 h post-infection [51]. This rapid invasion kinetics explains the earlier onset of mortality in our immersion group. Similarly, research on *Edwardsiella ictaluri* in channel catfish demonstrated that skin abrasion sites are rapidly colonized by pathogens, leading to significantly faster development of systemic infection and higher mortality rates compared to fish with intact skin [52]. These findings support our observation that the extensive skin surface area of tadpoles, when directly exposed to high concentrations of *Pseudomonas* sp. LBK2 in the water, provides numerous potential entry sites for rapid bacterial invasion. In contrast, the feeding route requires pathogens to survive the hostile environment of the gastrointestinal tract, including low pH, digestive enzymes, bile salts, and competing microbiota, before they can adhere to and invade the intestinal epithelium. This additional barrier creates a significant temporal delay in the establishment of systemic infection. Research on virulent *Aeromonas hydrophila* in channel catfish has shown that feeding status modulates immune responses and disease outcome, with fed fish exhibiting more severe gastrointestinal lesions characterized by epithelial necrosis, hemorrhage, and edema [53]. However, even with these lesions, the progression to mortality still requires time for the pathogen to traverse the intestinal barrier, multiply, and disseminate systemically. This aligns with our observation that mortality in the feeding group was delayed until day 3 post-infection.

Bacterial pathogens are common in aquaculture and cause significant losses to the aquaculture industry [54]. Comprehensive measures should be taken to effectively prevent and control these diseases, encompassing both prevention and treatment. On the prevention side, regular disinfection, water quality improvement, and rational use of aerators can be implemented. On the treatment side, external antibiotics can be used to eliminate pathogens, while internal medications can enhance the resistance of aquatic organisms [55]. For bacterial pathogens, antibiotic treatment is the most widely used and rapidly effective method. Here, the drug susceptibility test indicated that pathogen LBK2 was highly sensitive to polymyxin, neomycin, florfenicol, amikacin, ampicillin, doxycycline, enrofloxacin, ceftriaxone, and rifampicin. Among these antibiotics, rifampicin showed the best inhibitory effect against pathogen LBK2. Rifampicin is confirmed to exert its effect by inhibiting bacterial RNA polymerase [56,57], and previous studies have documented that rifampicin could inhibit *Pseudomonas* sp. in *Lates calcarifer* [58]. It should be noted that rifampicin exhibits certain toxicity and has been classified by the World Health Organization’s International Agency for Research on Cancer as a carcinogen. It is important to clarify that our identification of rifampicin as the most effective antibiotic in vitro serves primarily as a scientific reference for understanding the pathogen’s susceptibility profile rather than a direct recommendation for widespread clinical use in aquaculture. The primary value of this finding lies in demonstrating that strain LBK2 is highly sensitive to multiple antibiotics, providing a range of therapeutic options that can be selected based on safety, regulatory approval, and practical considerations.

## 5. Conclusions

The current study suggested that LBK2 *Pseudomonas* sp. is the pathogen of perforation disease in giant spiny frog tadpoles. This species possesses multiple virulence genes but is sensitive to many antibiotics, such as enrofloxacin, and doxycycline. These results provide a theoretical basis for the prevention and treatment of abdominal perforation disease in the tadpoles of the giant spiny frog. We acknowledge that this study has certain limitations that should be considered when interpreting the results. Notably, the pathogen isolation was based on only three diseased tadpoles collected from a single farm in Fuzhou City, Jiangxi Province, China. However, it is important to consider the context of this disease outbreak. The acute infection occurred in a commercial farming operation where affected tadpoles exhibited highly consistent and characteristic symptoms, including lethargy, anorexia, skin decay, and abdominal perforation. In such emergency situations, rapid intervention is critical to minimize economic losses. Therefore, the primary objective was to swiftly identify the causative agent to facilitate the timely development of control strategies. The three tadpoles selected for sampling were representative of the affected population, displaying the full spectrum of typical clinical signs.

## Figures and Tables

**Table 1:** The primers used in this study.

Genes	Sequences of Primers (5′~3′)		Amplification Fragment Size/Bp
16S rRNA	27F	AGAGTTTGATCCTGGCTCAG	1500
	1492R	GGTTACCTTGTTACGACTT	
lip	F	ATCTTCTCCGACTGGTTCGG	382
	R	CCGTGCCAGGACTGGGTCTT	
ompAI	F	GACGATATCATGATGAAAATGGCTCTT	1026
	R	GCGAAGCTTTTACTTCTGAACTTCTTG	
ompAII	F	GCTGAATTCATGAAACTCAAAATGGCTC	1001
	R	GCGAAGCTTTTACTGTTGTACTTGC	
fla	F	TCCAACCGTYTGACCTCw	608
	R	GMYTGGTTGCGRATGGT	
gcaT	F	CTCCTGGAATCCCAAGTATCAG	237
	R	GGCAGGTTGAACAGCAGTATCT	
alt	F	CCACCGGTATCGAACTTGAT	819
	R	CCAGACGGTGACGAAGT	
act	F	TTGATICCAGACGGTGACGAAGT	623
	R	CAGCCTTGTAGAGCTCGATCT	
ast	F	GTCAGCGACAGCTICTTCAT	354
	R	GTCAGCGACAGCTTCTTCAT	

## Data Availability

The original contributions presented in this study are included in the article/Supplementary Materials. Further inquiries can be directed to the corresponding author.

## Supplementary Materials

The following supporting information can be downloaded at: https://www.mdpi.com/article/10.3390/microorganisms14051016/s1, Figure S1: Colony Morphology and Gram Staining of LBK2; Figure S2: LBK2 identification Electrophoresis Image; Figure S3: Partial Results of Drug Sensitivity Test.
