# Supplementary material for: Isolation, Identification, and Drug Susceptibility Testing of the Pathogen Causing Perforation Disease in Giant Spiny Frog Tadpoles (Quasipaa spinosa)
Source: Microorganisms. 2026 Apr 30;14(5):1016. doi: 10.3390/microorganisms14051016 (PMC13209465; doi:10.3390/microorganisms14051016)
Supplement: Supplementary file 1 [file microorganisms-14-01016-s001.zip › microorganisms-4202023-supplementary.pdf]

Appendices

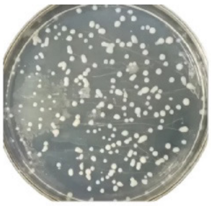

a.Bacterial colonies in LB medium

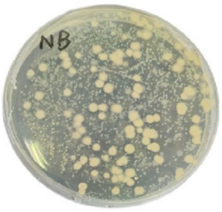

b.Bacterial colonies in NB medium

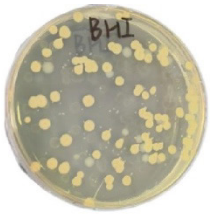

c.Bacterial colonies in BHI medium

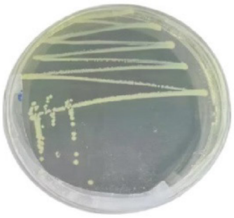

d.Purified LBK2

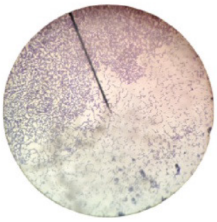

e.LBK2 after Gram staining

Figure S1. Colony Morphology and Gram Staining of LBK2

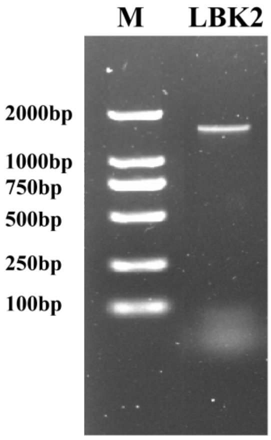

Figure S2. LBK2 identification Electrophoresis Image

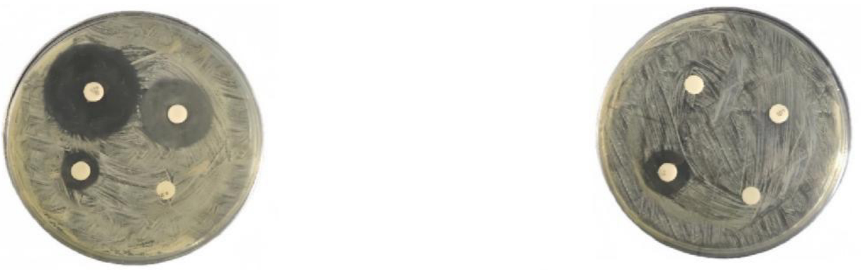

a. Cefotaxime, Enrofloxacin, etc

b.Trichloroisocyanuric acid and rifampicin drugs

Figure S3. Partial Results of Drug Sensitivity Test.
